# Characterizing the Spatiotemporal Distribution of Three Native Stink Bugs (Hemiptera: Pentatomidae) across an Agricultural Landscape

**DOI:** 10.3390/insects12100854

**Published:** 2021-09-22

**Authors:** Erin E. Grabarczyk, Ted E. Cottrell, Glynn Tillman

**Affiliations:** 1 Southeast Watershed Research Laboratory, USDA-ARS, Tifton, GA 31793, USA; 2 Southeastern Fruit and Tree Nut Research Laboratory, USDA-ARS, Byron, GA 31008, USA; Ted.Cottrell@usda.gov

**Keywords:** *Euschistus servus*, *Euschistus tristigmus*, *Chinavia hilaris*, SADIE, pheromone-baited trap, red–blue plot, non-crop habitat

## Abstract

**Simple Summary:**

Stink bugs (Hemiptera: Pentatomidae) are highly mobile pests that forage on, and damage, a variety of crops. Habitats that surround farms, such as forests, wetlands, and pastures may play a role in the location of stink bugs and their movement into crop fields. Here, stink bugs were trapped weekly across an 18 km^2^ agricultural landscape, and we characterized their distribution, as well as patterns of aggregation by habitat. Brown stink bugs (*Euschistus servus*) were most often captured in crop fields and the timing of aggregations often corresponded to food availability. Dusky stink bugs (*Euschistus tristigmus*) were primarily captured in forest, and only occasionally in crop fields. Green stink bugs (*Chinavia hilaris*) were found in both crop fields and non-crop habitat. Control of stink bugs may require management plans that consider movement not only within crop fields, but also the surrounding habitat.

**Abstract:**

Stink bugs (Hemiptera: Pentatomidae) are polyphagous pests that cause significant economic losses to a variety of crops. Although many species have been documented to aggregate within agricultural fields, much less is known regarding the timing and distribution of adults and nymphs within and between surrounding non-crop habitat. Therefore, we explored the spatiotemporal distribution of *Euschistus servus* (Say), *Euschistus tristigmus* (Say), and *Chinavia hilaris* (Say), three species of North American origin, and examined whether distribution patterns varied between species according to habitat. Stink bugs were monitored weekly for three years within an 18 km^2^ grid of pheromone-baited traps. We tested whether habitat affected distribution patterns, used spatial analysis by distance indices (SADIE) to identify aggregations, and visualized distributions with interpolated maps. Overall, *E. servus* adults were captured in crops, whereas *E. tristigmus* adults and nymphs were mainly captured in forests. Accordingly, distribution patterns of *E. tristigmus* were relatively stable over time, whereas aggregations of adult *E. servus* varied over space, and the timing of aggregations reflected the phenology of major crops. *Chinavia hilaris* was most often captured in forest, followed by crop habitat. Pest management strategies for stink bugs may require an area-based management approach that accounts for movement in agricultural fields and surrounding habitat.

## 1. Introduction

The distribution and dispersal of stink bugs (Hemiptera: Pentatomidae) in agricultural landscapes varies over space and time [1,2,3,4]. For these highly mobile polyphagous pests, such patterns are in part driven by the location of plants that are suitable reproductive hosts and food sources. This results in individuals that disperse from one host plant to the next, often forming high-density aggregations that tend to occur within crop field edges [5]. Although many species have been documented to aggregate in major crop hosts, much less is known regarding their distribution patterns within and between habitats that surround agricultural fields. Forest, pasture, and wetland habitats may provide stink bugs a refugia from insecticides [6] or a place to overwinter [7], as well as offer additional non-crop food resources, which may also serve as reproductive hosts [8]. Therefore, understanding how non-farmed habitats near crop fields affect the distribution of stink bugs can inform economically and ecologically sustainable pest management strategies.

The spatiotemporal distribution and dispersal patterns of stink bugs across agricultural landscapes may differ not only between species, but also according to stage of development. The mobility of adult stink bugs differs from that of nymphs; adults can fly from one location to another, whereas nymphs walk [9]. The speed at which adults and nymphs disperse between host plants may depend on several factors. For example, the speed at which *Halyomorpha halys* (Stål) nymphs walk varies by instar, but abiotic factors, such as ambient temperature, also affect walking speed [9]. In general, female stink bugs oviposit egg masses on plant leaves, where early instars then feed while older instars disperse to feed on leaves, stems, and fruit [10,11,12]. Accordingly, the presence of early instars in traps or near a given plant species can be used to estimate where females oviposit egg masses and to identify plants that may serve as reproductive hosts. Ultimately, by investigating the dispersal of stink bugs by life stage (i.e., adults and nymphs) in a diversity of habitats, we can better understand the factors that underlie variation in seasonal distribution patterns.

In southeastern US agroecosystems, three common native stink bug species include *Euschistus servus* (Say), *Euschistus tristigmus* (Say), and *Chinavia hilaris* (Say). In this region, *Euschistus* spp. colonize in, and forage on, corn, peanut, and cotton; all serve as good hosts [13]. *Euschistus tristigmus* is a major stink bug pest of cotton, and the congeneric species *E. servus*, *E. quadrator* (Rolston), and *E. ictericus* (L.) are also pests of this crop [13]. Moreover, *Euschistus* spp. oviposit on peanut, and subsequent nymphs develop on peanut leaves, but they are not currently considered economic pests of this crop [13]. For *C. hilaris*, cotton is also a good host plant; however, peanut is a poor one, and corn is typically never colonized by this species [13]. Additional reports from other locations in the state of Georgia agree with these findings (e.g., [14,15]). In addition, non-crop hosts that grow in forest can serve as a source of stink bugs that later disperse into crops. Three common non-crop sources in southeastern forests include black cherry (*Prunus serotina* Ehrh.), elderberry [*Sambucus nigra* subsp. *canadensis* (L.) R. Bolli], and mimosa (*Albizia julibrissin* Durazz.). Black cherry is an early-season reproductive host plant and food source primarily for *C. hilaris*, but occasionally serves as a host for *E. servus* and *E. tristigmus* [8]. Elderberry is a mid-season food source and reproductive host for *C. hilaris*, *E. servus*, and *E. tristigmus* [16], and mimosa is a mid-season food and reproductive host for *C. hilaris* [17].

Several studies have shown that pyramid traps baited with commercially available lures of aggregation pheromone attract specific stink bug species, and effectively capture adults and nymphs in the field. The aggregation pheromone of *Euschistus* spp., methyl (2*E*,4*Z*)-decadienoate (MDD), attracts *E. servus* and *E. tristigmus* in the field [18,19,20,21,22,23]. *Chinavia hilaris* is cross-attracted to the aggregation pheromone of *Plautia stali* Scott, methyl (2*E*,4*E*,6*Z*)-decatrienoate (MDT) under field conditions [24], and pyramid traps baited with this pheromone capture *C. hilaris* in peanut and cotton fields [23]. Stink bug nymphs also are drawn to traps with the synthetic aggregation pheromones attractive to their species [23,25,26]. Therefore, pheromone-baited traps can be used to monitor both adult and nymphal populations.

The objective of this study was to explore the effects of habitat on native stink bug distributions and to examine whether spatiotemporal patterns differ between adults and nymphs across a diverse agricultural landscape. We monitored an 18 km^2^ grid of pheromone-baited traps and collected stink bugs weekly over a three-year period and explored the relative effects of major crops, as well as non-crop habitats, on distribution patterns.

## 2. Materials and Methods

### 2.1. Field Collection

**Figure 1:**
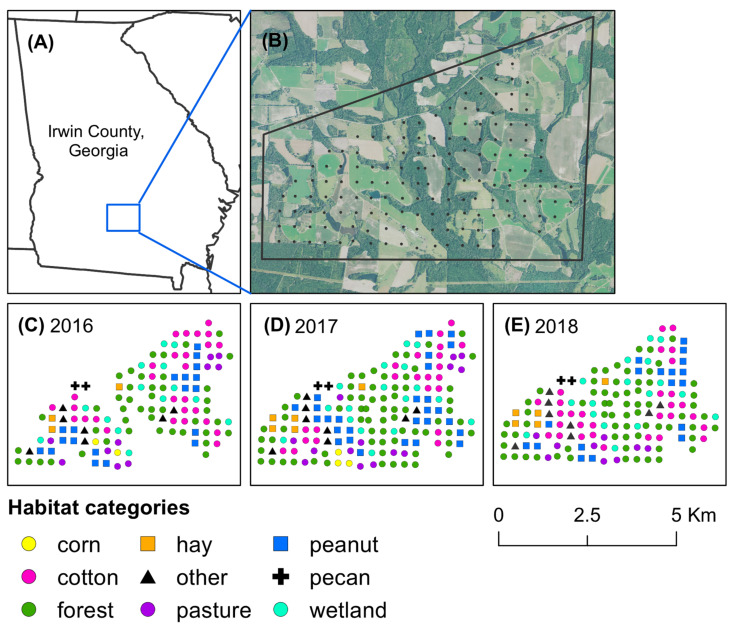
Map of Irwin County, Georgia, USA, and habitat categories at pheromone-baited traps. The study took place in (**A**) Irwin County, Georgia, where stink bugs were captured across (**B**) an 18 km^2^ agricultural landscape. Habitats at each trap site were categorized for (**C**) 2016, (**D**) 2017, and (**E**) 2018, based on predominant land use type.

From 2016–2018, stink bugs were trapped across an 18 km^2^ landscape in Irwin County, Georgia, USA (Figure 1). The two major crops within the study area included cotton and peanut, as well as smaller areas of cultivated corn and pecan. Stink bug adults may feed on leaves and stems of plants, but they primarily feed on fruit (excluding peanut). Thus, timing of crop phenology was noted for corn from very young to mature ears, cotton from first flower through mature bolls, and peanut from young foliage to harvest within each field with at least one baited trap. Habitats that surrounded agricultural fields included forest (both natural woodlands and pine), wetland, and water, as well as human-built infrastructure such as roads, houses, and farm-related buildings, and equipment. Hardwood tree species in forest and wetland included mockernut hickory (*Carya tomentosa* Sarg.), pignut hickory (*Carya glabra* Miller), pecan (*Carya illinoinensis* (Wangenh.) K. Koch), walnut (*Juglans nigra* L.), sweetgum (*Liquidambar styraciflua* L.), oak (*Quercus* spp.), red maple (*Acer rubrum* L.), tulip poplar (*Liriodendron tulipifera* L.), dogwood (*Cornus florida* L.), sycamore (*Platanus occidentalis* L.), red bud (*Cercis canadensis* L.), elm (*Ulmus americana* L.), and cypress (*Cupressus* spp.). The primary pine species in forests were loblolly (*Pinus taeda* L.) and slash (*Pinus elliottii* Engelm). Non-crop stink bug hosts that grew in forests included black cherry, elderberry, and mimosa. All three non-crop hosts located within 30 m of a pyramid trap were identified, and the timing of plant phenology was noted for each species from very young fruit until fruit maturity.

Stink bugs were captured with yellow pheromone-baited pyramidal traps. Across the study landscape, traps were set in a grid and separated by approximately 300 m (Figure 1B). For each trap, the predominant type of habitat was categorized as forest, wetland, corn, peanut, cotton, hay field, pecan orchard, livestock pasture, and other (roadside near a field edge, edge of a pond, or fallow field) for each year of the study based on ground-truthing of the area (Figure 1C–E). Each stink bug trap consisted of an insect-collecting device made from a 2.8 L clear plastic PET jar (United States Plastic Corp., Lima, OH) with a screw-cap lid (10.2 mm in diameter) seated atop a 1.22 m tall yellow pyramid base [19]. The insect-collecting device was baited with a lure of the aggregation pheromone of *Euschistus* spp. (MDD) and a lure of the aggregation pheromone of *P. stali* (MDT). The MDD pheromone was purchased from Bedoukian Research, Inc. (Danbury, CT, USA), and lures with the pheromone were produced following the procedures in Cottrell and Horton [27]. MDT lures were purchased from AgBio, Inc. (Westminster, CO, USA). An insecticidal ear tag (10% λ-cyhalothrin and 13% piperonyl butoxide) (Saber extra insecticide ear tags, Sagebrush Tags, De Smet, SD, USA) was also placed in each device to decrease the likelihood of stink bug escape [28]. Once per week, lures were replaced and stink bugs captured in traps were transferred to resealable bags and stored at the USDA Southeast Watershed Research Unit in Tifton, GA. Identification of adults was based on a taxonomic key [29], and identification of nymphs was based on several years of experience rearing stink bugs in the laboratory. During reproductive diapause, the abdomen of *E. servus* tends to change from green to reddish brown [30], therefore reddish-brown *E. servus* and *E. tristigmus* adults captured in traps were considered to be overwintering.

### 2.2. Data Analysis

We calculated the percent relative abundance of adult and nymphal stink bugs captured in pheromone-baited traps (Supplementary Materials, Table S1). If the relative abundance was at least 20% for adults and/or nymphs for a given species, then that species was included in analyses. R program software was used to fit generalized linear mixed effect models (package: lme4, function: glmer [31]) to analyze the relative effects of habitat on counts of the most prevalent adults and nymphs captured (logit = Poisson, family = link). Our analysis consisted of six separate models, which included the number of *E. servus*, *E. tristigmus*, and *C. hilaris* adults; *E. servus*, *E. tristigmus*, and *C. hilaris* nymphs captured each week in pheromone-baited traps as our response variable. Habitat (three levels; row crop, forest–wetland, and other) was included as our fixed effect, and year of collection as well as trap site nested within Julian date were included as random effects. To assess model adequacy, we used residual plots and checked for overdispersion. For each model, we obtained estimated marginal means (package: emmeans, function: pairs; [32]) to assess differences in the number of stink bugs captured between the three types of habitat and considered counts within habitats to differ significantly when *p* < 0.05.

We used spatial analysis by distance indices (SADIE; [33]) to analyze weekly aggregation patterns of stink bug adults and nymphs over the three-year collection period (R program software; package: epiphy, function: sadie; [34]). In R, *I_a_* was calculated for each week sampled, which, if significant, indicated that stink bugs were clustered in space during that sample period. *I_a_* was considered significant if *P_a_* was <0.05 [33,35]. For weeks with a significant *I_a_* (i.e., overall clustering), we then calculated local gap and patch indices for each trap site to determine the specific trap location where stink bugs clustered in the landscape. For each trap site, weekly values of >1.5 suggest significant aggregations, or a large number, of stink bugs at that specific location, whereas weekly values of <−1.5 suggest gaps in stink bug distributions [33,35]. To visualize weekly patterns of significant clustering at trap sites based on the SADIE analysis, we interpolated red–blue maps in ArcMap (version 10.5, ESRI, Redlands, CA, USA) based on local trap site gap and patch indices with the inverse distance weighting tool (IDW; power = 2, variable = 20 points). Red interpolated areas suggest significant aggregations or patches, and blue interpolated areas indicate significant clusters as gaps in stink bug distributions [33]. In addition to interpolations, trap sites were symbolized on maps in red if the local trap site cluster index was >1.5, or a significant aggregation, and in blue if the index was <−1.5, or a significant gap. Although SADIE cluster analysis identifies where significant aggregations occur in the landscape, this type of analysis does not indicate the number of individuals that form aggregations [36]. However, density data can be used to understand the magnitude of counts that make up significant aggregations, as well as display broader seasonal distribution patterns of the general population. Therefore, we also visualized spatiotemporal patterns based on count data (i.e., density) for each species, by generating interpolated map estimates for each stink bug species in ArcMap for each trap location by week and year (IDW; power = 2, variable = 20 points). For density maps, weeks were excluded from the map image if no stink bugs were captured during that sample period (Supplementary Materials, Figures S1–S3).

## 3. Results

Over the three-year study, 80,073 stink bugs representing 18 species were captured. The most prevalent adult stink bug species was *E. servus*, followed by *E. tristigmus*, and *E. ictericus* (Supplementary Materials, Table S1). The most prevalent nymph species was *E. tristigmus*, followed by *E. servus*, and *C. hilaris* (Supplementary Materials, Table S1). Mostly adults (92.2%) were captured. The number of adult *E. servus* differed significantly (*F* = 399.3, df = 2, *p* < 0.0001) by habitat; adults were most often captured in row crops, followed by other habitats, with fewer individuals captured in forest and wetland (Table 1). The number of *E. servus* nymphs captured was not significantly influenced by habitat (*F* = 0.4, df = 2, *p =* 0.2). Trap capture was significantly higher for *E. tristigmus* adults (*F* = 1021.3, df = 2, *p* < 0.0001) and nymphs (*F* = 247.6, df = 2, *p* < 0.0001) in forest and wetland habitat compared to row crops, followed by other habitats (Table 1). The number of *C. hilaris* adults did not differ by habitat, and overall, counts of adults were low across habitats (Table 1). For nymphs, trap capture was significantly different (*F* = 0.7, df = 2, *p* = 0.005) by habitat; more nymphs were captured in forest and wetland compared to other habitats, but the number of nymphs did not differ between forest and wetland habitat and row crops.

The spatiotemporal distribution patterns of adult and nymphal stink bugs varied by year and by species. In general, the number of *E. servus* adults in traps peaked during mid-to-late summer and again during the fall, which represents a generation of stink bugs that developed in crops and an overwintering generation, respectively. This corresponds with high densities of *E. servus* individuals captured in peanut (July–August) and in cotton following defoliation and harvest (October; Supplementary Materials, Figure S1). The number of *E. tristigmus* captured was more consistent over time compared to *E. servus* and was typically distributed near traps in forest habitat near crop field edges (Supplementary Materials, Figure S2). Overall, the number of *C. hilaris* adults captured in traps was consistently low. However, if captured, adult and nymph *C. hilaris* tended to be found in forest, wetland, and cotton (Supplementary Materials, Figure S3).

**Figure 2:**
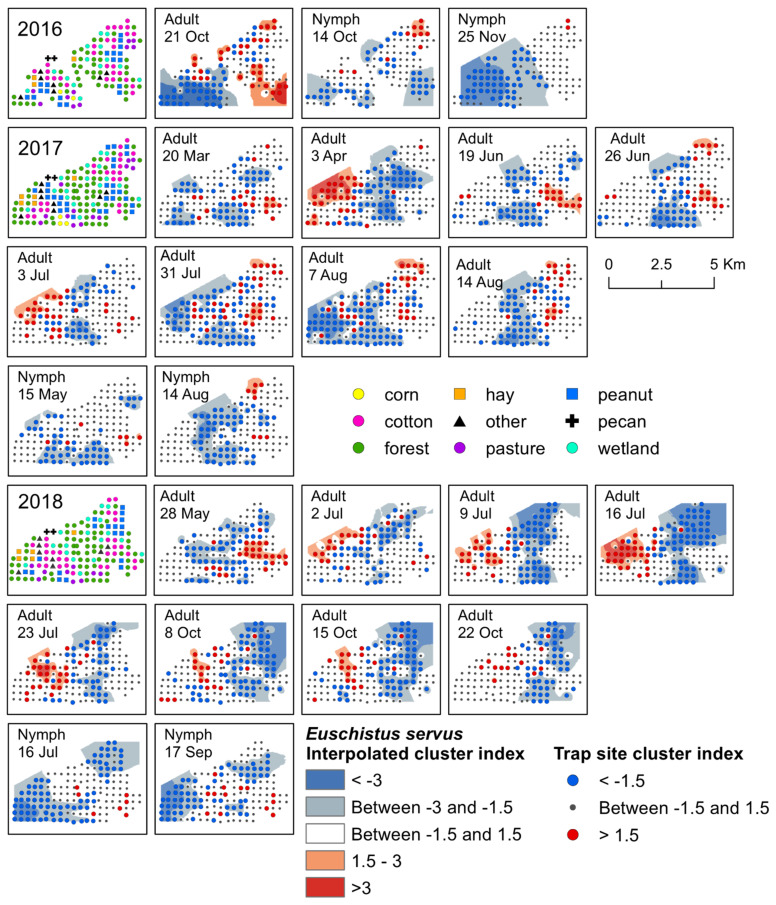
Red–blue plots based on interpolation of the cluster index for *E. servus* adults and nymphs from 2016–2018. Red areas (>1.5) indicate significant clustering in aggregations in distributions; blue areas (<−1.5) indicate significant clustering as gaps in distribution.

Significant clusters of *E. servus* adults were detected in each year of the study, primarily during July, and again in October. In general, the location of the aggregations shifted each year following change in host crop phenology. For example, aggregations in peanut during July and August moved into cotton just prior to, and following, harvest and the surrounding habitat by October (Figure 2). Early-season clusters of adult *E. servus* were detected in 2017 and 2018. In April 2017, adults aggregated in a variety of habitats including a young pecan orchard, forest, livestock pasture, in forest adjacent to a peanut field, within a peanut field, along a road near a cotton field, in wetland habitat adjacent to a peanut field, in forest adjacent to a corn field, and in a fallow field (Figure 2). Another early-season cluster was detected in May 2018; adults aggregated along cotton field edges and in cotton fields, in forest and wetland, and in a peanut field, as well as in other habitats, including a livestock pasture and along a road next to corn (Figure 2). Significant clusters of adult *E. servus* were detected from mid-June until mid-August 2017 (Supplementary Materials, Table S2). During this time, aggregations occurred primarily within cotton and peanut fields, as well as along field edges adjacent to these crops near late-season black cherry and elderberry (Supplementary Materials, Table S3; Figure S2). Significant clusters of adults were detected mid-season during each week of July 2018 (Supplementary Materials, Table S4). Aggregations occurred on the western portion of the landscape, and gaps were located towards the center of the landscape (Figure 2). The specific habitats where adults aggregated included a young pecan orchard, along cotton field edges, cotton and peanut fields, forest, hay field, at an interface between cotton and peanut, and in forest near each of the three main crops and a soybean field (Figure 2). During this time (i.e., mid-to-late season), black cherry and elderberry fruit were available on plants of these non-crop hosts located near several traps. In mid-October 2016, *E. servus* adults aggregated in harvested fields or field edges previously planted in cotton, peanut, and hay, as well as forest and wetland habitat adjacent to the harvested crops (Figure 2). Adults aggregated for a three-week period during October 2018 (Supplementary Materials, Table S4). Similarly, these clusters occurred within post-season cotton and peanut, as well as along forest edge adjacent to fields of both crops. From 2016–2018, adults in overwintering condition were most often captured in previous cotton fields, forest, and wetland (Supplementary Materials, Figure S1). All significant aggregations of adult *E. servus* in trap captures with overwintering individuals occurred following peanut harvest and close to, or following, cotton harvest. The habitats where these aggregations occurred included cotton and peanut, forest, and wetland adjacent to both crops, wetland and forest, and a young pecan orchard.

The earliest detection of *E. servus* nymphs occurred during the month of March in traps located in forest, wetland, cotton, and peanut. Early-season clusters of nymphs were detected in mid-May 2017; during this week, aggregations of nymphs occurred at a forest trap next to a cotton field and in a cotton and peanut field, all of which were in close proximity to fruiting black cherry trees (Figure 2). Later, clusters were detected for a week in mid-August 2017, where aggregations of nymphs were detected in peanut fields and along the edge of peanut fields, and in wetland near a cotton field edge (Figure 2). During this same week in August, nymphs also aggregated near trap sites with mimosa and elderberry plants with late-season fruit located along the edge of a cotton field and a peanut field (Supplementary Materials, Table S3). Gaps on that date occurred across the central portion of the landscape in a variety of habitats (Figure 2). During 2018, *E. servus* nymphs clustered for a week in mid-July, with aggregations occurring in cotton, as well as in forest next to cotton, and corn field edges. During that week, corn plants had fruit (i.e., ears) and fruiting elderberry plants were present near corn traps. In mid-September 2018, aggregations occurred at traps located in cotton and peanut and forest adjacent to corn and cotton (Figure 2). At the time, peanut and cotton were the only available crop food sources near traps with aggregations (Supplementary Materials, Table S4). Later season, significant clusters of *E. servus* nymphs were detected in October and November 2016, with gaps occurring in wetland, forest, peanut, pasture, corn, and cotton in October and in a variety of habitats across the western half of the landscape in November (Figure 2). Aggregations of nymphs were detected mainly in harvested cotton but also in forest, a peanut field, and a livestock pasture (Figure 2).

**Figure 3:**
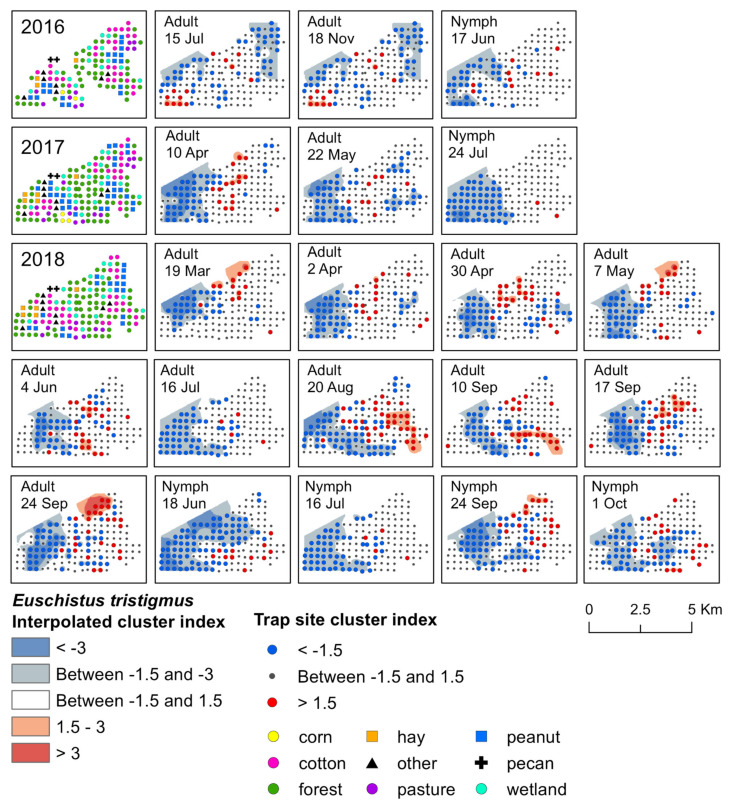
Red−blue plots based on interpolation of the cluster index for *E. tristigmus* adults and nymphs from 2016–2018. Red areas (>1.5) indicate significant clustering in aggregations in distributions; blue areas (<−1.5) indicate significant clustering as gaps in distribution.

Significant clusters of adult *E. tristigmus* were detected during each year of the study. Although the timing was less consistent than *E. servus*, the location of clusters was relatively stable (Figure 3). Aggregations of adults were found near the center of the study landscape, where a large area of forest and wetland, interspersed with some crop fields, was located. During 2016, two significant weeks of clustering were detected in June and early July (Supplementary Materials, Table S2). Aggregations occurred at traps near black cherry trees with mature fruit. Early-season aggregations were detected in 2017; adult *E. tristigmus* aggregated in mid-April, primarily in forests, as well as forest habitat on the edge of cotton and peanut fields (Supplementary Materials, Figure S2).

In 2016, significant aggregations of adults were detected in mid-July and later in mid-November (Figure 3). Aggregations at both time points were located primarily in forest habitat. However, some clusters were detected in variable landscapes, including wetland habitat, as well as cotton and peanut adjacent to forest edge (Figure 3). Overall, mid-season, adult *E. tristigmus* aggregated at a total of five trap sites that had non-crop host plants with fruit. First, in two traps in both forest and wetland with mid-season black cherry in mid-July 2016, and again in a wetland trap in close proximity to elderberry with late-season fruit in mid-August 2018. Significant aggregations at traps that contained overwintering *E. tristigmus* adults in 2016 occurred mainly in forest and wetland habitat, but also in harvested cotton.

The earliest detection of *E. tristigmus* nymphs occurred during the month of June in traps located in forest and wetland. Two significant clusters of *E. tristigmus* nymphs were detected over the entire three-year study period. The first aggregation occurred in mid-June 2016 at traps located in forest habitat, as well as forest habitat along corn, cotton, and peanut field edges (Figure 3). In 2018, the second cluster was also detected in mid-June, located in the same type of habitat (i.e., forest and forest–agricultural field edge; Supplementary Materials, Table S4). Similar to adults, *E. tristigmus* nymphs were generally found in forest or in habitats located near forest edges.

**Figure 4:**
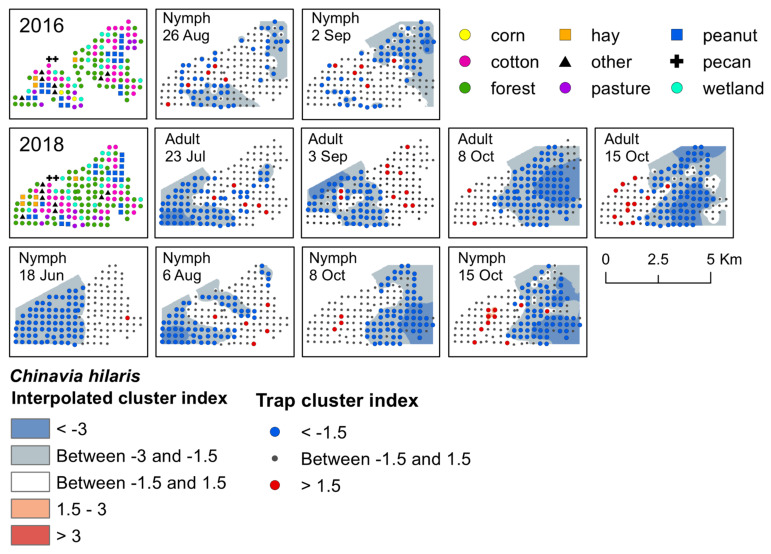
Red−blue plots based on interpolation of the cluster index for *C. hilaris* adults and nymphs from 2016–2018. Red areas (>1.5) indicate significant clustering in aggregations in distributions; blue areas (<−1.5) indicate significant clustering as gaps in distribution.

Significant clusters of *C. hilaris* nymphs were detected in both 2016 and 2018, and in 2018 for adults. One early-season cluster was detected; in June 2018, nymphs aggregated in a cotton field that was near fruiting black cherry (Figure 4). Gaps occurred on the western half of the landscape. During late-season 2016, *C. hilaris* nymphs aggregated at the end of August and beginning of September in a fruiting cotton field, as well as in forest, along peanut field edges, and in a wetland habitat with late-season elderberry that was adjacent to a cotton field edge (Figure 4). They were also clustered in a wetland habitat containing non-fruiting black cherry next to a field edge of late-season cotton. In August 2018, aggregations occurred at traps located in wetland and forest habitat next to cotton and corn field edges near fruiting elderberry (Figure 4). From early to mid-October, aggregations of nymphs were found primarily in harvested cotton, with a few clusters occurring in forest and wetland next to cotton and peanut field edges. Two significant aggregations were detected for two consecutive weeks in a harvested peanut site surrounded by forest with non-fruiting black cherry trees. During this time, gaps shifted towards the eastern half of the landscape (Figure 4).

Overall, the number of *C. hilaris* adults captured in traps was low (approximately one adult to every three nymphs captured), and oftentimes too few adults were captured by week to analyze the data for significant aggregations. However, if captured, both adults and nymphs tended to be found at the same trap locations. Adult *C. hilaris* clustered in 2018 during mid-July, early September, and for two weeks in October (Supplementary Materials, Table S4). In July, adults aggregated in a wetland habitat near a cotton field edge that contained mid-season elderberry, forest, and a field edge of very young peanut (Supplementary Materials, Table S4). In September, adults aggregated in cotton. They also clustered in a few peanut fields, but no nymphs were ever detected in these fields. All aggregations in October occurred at traps that contained overwintering adults. The habitats where aggregations occurred included harvested cotton and peanut, a young pecan orchard, and forest. Similar to nymphs, gaps occurred on the western portion of the landscape in July and September, and shifted towards the eastern portion of the landscape in October (Figure 4).

## 4. Discussion

The spatiotemporal distribution of stink bugs across a diverse, heterogenous landscape varied among species, between adults and nymphs, and by habitat. Stink bugs colonize host plants throughout the growing season, oftentimes dispersing in response to deteriorating suitability of the current host, as new host plants become available [1,3,4,14,37]. Here, *E. servus* adults were most often captured in known host crop habitats, including corn, cotton, and peanut, and aggregations of adults shifted across the landscape following the phenology of crop development. Counts of *E. servus* nymphs did not differ between habitats, but significant aggregations were detected in cotton, peanut, and forest. Similar to distribution patterns in Georgia peach orchards, *E. tristigmus* adults and nymphs were typically captured in forest and wetland habitat [38]. The specific trap location where *E. tristigmus* clustered varied, but the type of habitat (i.e., forest and wetland) where this species dispersed remained stable across seasons and over each year of the study. Few *C. hilaris* adults were captured in traps, and nymphs of this species were more common. *Chinavia hilaris* nymphs tended to aggregate in cotton, especially in traps located near non-crop host plants, as well as in diverse habitats including in forest edge adjacent to cotton and peanut fields and in forest and wetland habitat. Together, density maps and SADIE show that the majority of stink bugs were captured and aggregated in predominantly in two crops, cotton or peanut, or forest and wetland habitat. Fewer stink bugs were captured or aggregated in other types of habitats, which included pasture, herbaceous wetland, fallow fields, and areas near water.

For stink bugs, the availability of crop and non-crop host plants, under favorable climatic conditions, play an important role in their distribution and aggregation patterns. The timing of *E. servus* aggregations, the most prevalent stink bug captured, was consistent across years and corresponded to the availability of food sources. In the southeastern US region, corn, peanut, and cotton all serve as crop hosts for *E. servus* [13]. Corn is the first major early-season crop and stink bug host plant to mature [39]. Although corn was grown during each year of the study, it was not the predominant crop, and significant clusters of adult *E. servus* during the time when corn plants had mature ears were uncommon. However, in the southeast United States, particularly in areas where corn is dominant in the landscape, it may be an early season source of *E. servus* populations that later disperse to nearby cotton and peanut fields [39]. The largest peaks in *E. servus* population density, as well as the likelihood of significant aggregations at trap sites, typically corresponded to periods when cotton fruit (i.e., bolls) and peanut plants were available in fields. A previous study showed that the composition of host crops, especially corn and peanut, within a landscape had a greater positive influence on *E. servus* reproduction than the proportion of non-crop habitat, including forest, pasture, and non-crop hosts [40]. Nonetheless, in this study, *E. servus* adults and nymphs aggregated at traps in field edges of each crop that were located near early-season fruiting black cherry and mid-season elderberry in forests. Elderberry fruit often begin to deteriorate around the same time that cotton and peanut become available, which may facilitate adult dispersal from elderberry into crop fields. In an earlier study, when elderberry was established in close proximity to crop hosts, such as cotton or peanut, the number of stink bugs that dispersed into crop fields was higher, compared to fields without elderberry [16]. Later in the season, high densities of adults, many of which were in overwintering condition, were present in cotton fields following defoliation and harvest. When high-density aggregations of adults were detected in other types of habitats, they were most often found during the spring and summer months in traps located in active grain (hay) fields. *Euschistus servus* often colonize other grain crops such as wheat, corn, and soybean, where their populations increase [37,39]. Thus, grain crops are likely an important source of *E. servus* in southeastern agroecosystems.

Although *C. hilaris* utilizes cotton as a host crop, this species is rarely detected in corn and peanut [13]. When *C. hilaris* adults clustered in peanut, nymphs were never detected in traps, suggesting that adults flying through the fields were arrested by MDT pheromone. Moreover, black cherry, elderberry, and mimosa in forest habitat are significant non-crop hosts of *C. hilaris* in the southeast [8,16,17]. In this study, *C. hilaris* aggregated near cotton in traps close to black cherry and elderberry plants with mature fruit, which again coincided with cotton boll availability. In an earlier mark–recapture study, as elderberry fruit senesced and cotton bolls became available, stink bugs, especially *C. hilaris*, dispersed from elderberry into cotton [16]. Therefore, elderberry likely serves as a host plant for stink bug adults and nymphs that subsequently disperse into crop fields. Accordingly, edge-specific control measures may reduce the impact of non-crop sources near crop field edges.

In contrast to *E. servus* and *C. hilaris*, non-crop hosts within the forest likely play a major role in development and distribution of *E. tristigmus*. Certainly, adults aggregated near crop fields edges in close proximity to fruiting black cherry and elderberry, early- to mid-season non-crop hosts of this stink bug [8,16]. In addition, volunteer pecan in forest habitats may serve as a non-crop host of *E. tristigmus* later in the season. Both *E. tristigmus* and *E. servus* are known predominant pentatomid species that attack pecan in orchards [19,41,42]. In addition, a recent study suggests that *E. tristigmus* is a major stink bug species captured in pheromone-baited traps placed near volunteer pecan within forests next to crops (Tillman, unpublished data). Despite consistent high densities of *E. tristigmus* in forest and wetland, both adults and nymphs were occasionally captured within a cotton or peanut field interior. Aggregations within crop fields tended to coincide with the availability of food. Similar to adults, *E. tristigmus* nymphs were most often found in wetland and forest habitat, and occasionally along crop field edges. This suggests that adults likely oviposit in forest and wetland habitat, as well as along crop field edges. Aggregations of overwintering adults were most often found in wetland and forest habitat, but, in a few instances, were also found in cotton fields prior to and following crop harvest.

The influence of forest habitat on stink bug densities in crops has previously been investigated for native and invasive species. For native species, *E. servus* adults were more likely to colonize areas near forest edge than corn field interiors, and *C. hilaris* was typically more common near forest edge than in cotton or soybean field interiors [15]. In a later study, edge effects on dispersal of adults were detected for *E. servus* in corn next to forest, as well as for both *E. servus* and *C. hilaris* in cotton adjacent to forest [5]. In both corn and soybean, fields adjacent to forest consistently harbored significantly higher densities of the invasive brown marmorated stink bug, *H. halys*, than in fields adjacent to open habitats, buildings, and corn [43]. Common forest plants, such as black cherry, elderberry, and mimosa, support reproductive populations of native stink bugs [8,16,17]. Similarly, tree of heaven (*Ailanthus altissima* Swingle), princess tree (*Paulownia tomentosa* Baill.), and black cherry support high population densities of reproducing *H. halys* [12,44,45]. Thus, forests likely play an important role in serving as a source of both native and invasive stink bug populations that later colonize crops.

For pest management, identifying areas within the landscape that support consistently high densities of pests is important to assess the risk of outbreaks and to implement targeted control measures. Furthermore, such locations may share common features that vary over time, such as non-crop host plants with mature fruit. SADIE analysis of aggregations is one approach that has been used in a variety of contexts, and is commonly used to explore spatiotemporal distribution patterns of arthropods in agriculture systems [35]. For example, SADIE has been used to characterize the distributions of arthropod pests [4,46] and predators [47,48] and to identify overlapping aggregations between pests and their natural enemies [49,50]. While this approach is most often used to understand aggregation patterns within individual crop fields or farmscapes with multiple crops, few studies have investigated arthropod spatiotemporal aggregations in surrounding non-crop habitats, such as between crop fields [47,51]. Particularly for highly mobile polyphagous pests, such as stink bugs, narrowly focused analysis of aggregations within fields could underestimate the importance of the surrounding non-crop habitat on population dynamics [16]. To our knowledge, this is the first study that uses SADIE analysis to understand the spatiotemporal distribution of stink bugs in both crop fields and the surrounding non-crop habitats. Additional work is needed to understand how natural enemies of stink bugs overlap in space and time with these pests, and to determine the types of habitats where aggregations co-occur in agroecosystems.

## 5. Conclusions

Using pheromone-baited traps placed across a large landscape, we show that three native stink bugs aggregated in non-crop habitats as well as crop fields, and the observed patterns in crop fields tended to be species-specific, whereas early- and mid-season non-crop hosts in forests are likely important sources of stink bugs, regardless of species. Accordingly, pest management strategies for stink bugs may require taking an area-based approach that accounts for movement in agricultural fields and the surrounding habitat. Combined, we demonstrate that SADIE cluster analysis and interpolated density maps can be used to identify stink bug hot spots, as well as common features among host plants (i.e., non-crop host plants with mature fruit), according to species in a variety of habitats. In the future, this approach may be used for targeted stink bug management. For example, identifying areas of high-density aggregations may be used to aid IPM strategies such as planned parasitoid releases (reviewed by [52]) or the placement of physical barriers to deter dispersal into crop fields from non-crop sources [53,54,55]. An important next step will be to link local non-crop host plant availability with stink bug distribution patterns and other mobile pests in agroecosystems.

## Figures and Tables

**Table 1:** Mean ± SE number of adult and nymph *Euschistus servus* (Say), *Euschistus tristigmus* (Say), and *Chinavia hilaris* (Say) stink bugs captured in pheromone-baited traps by habitat.

	Row	Forest + Wetland	Other
Species	Mean ± SE	Mean ± SE	Mean ± SE
*E. servus* adult	7.61 ± 0.24 a	2.74 ± 0.07 c	3.27 ± 0.14 b
*E. servus* nymph	0.15 ± 0.001 a	0.16 ± 0.01 a	0.12 ± 0.01 a
*E. tristigmus* adult	0.62 ± 0.03 b	2.22 ± 0.05 a	0.35 ± 0.04 c
*E. tristigmus* nymph	0.09 ± 0.01 b	0.39 ± 0.02 a	0.07 ± 0.01 c
*C. hilaris* adult	0.07 ± 0.01 a	0.04 ± 0.004 a	0.01 ± 0.002 a
*C. hilaris* nymph	0.09 ± 0.01 ab	0.16 ± 0.02 a	0.01 ± 0.004 b

For each species by stage, mean ± SE followed by the same letter in the same row are not significantly different (estimated marginal means pairwise contrasts, *p* < 0.05).

## Data Availability

The data presented in this study are available in the article.

## Supplementary Materials

The following are available online at https://www.mdpi.com/article/10.3390/insects12100854/s1, Table S1: Percent relative abundance of stink bug species (Hemiptera: Pentatomidae) according to total number of adults and total number of nymphs captured in pheromone-baited traps from 2016–2018 in Irwin County, GA, USA, Table S2: Stink bug adult and nymph aggregation indices (Ia)a in Irwin County, GA, USA during 2016 based on SADIE spatial analysis, Table S3: Stink bug adult and nymph aggregation indices (Ia)a in Irwin County, GA, USA during 2017 based on SADIE spatial analysis, Table S4: Stink bug adult and nymph aggregation indices (Ia)a in Irwin County, GA, USA during 2018 based on SADIE spatial analysis, Figure S1: Interpolated maps based on annual trap capture that shows the distribution of the total number of Euschistus servus (nymphs and adults) captured from 2016–2018, Figure S2: Interpolated maps based on annual trap capture that shows the distribution of the total number of Euschistus tristigmus (nymphs and adults) captured from 2016–2018, Figure S3: Interpolated maps of C. hilaris distributions from 2016–2018.
