# Supplementary material for: Characterizing the Spatiotemporal Distribution of Three Native Stink Bugs (Hemiptera: Pentatomidae) across an Agricultural Landscape
Source: Insects. 2021 Sep 22;12(10):854. doi: 10.3390/insects12100854 (PMC8540503; doi:10.3390/insects12100854)
Supplement: Supplementary file 1 [file insects-12-00854-s001.zip › insects-1354999 - supplementary.pdf]

Supplemental Materials

Supplemental Materials Table S1. Percent relative abundance of stink bug species (Hemiptera: Pentatomidae) according to total number of adults and total number of nymphs captured in pheromone-baited traps from 2016 – 2018 in Irwin County, GA, USA.

Species	% Relative Abundance	
	Adults	Nymphs
Banasa euchlora (Stål)	0.03	0
Banasa dimidiata (Say)	0.07	0
Brochymena arborea (Say)	0.14	0.33
Chinavia hilaris (Say)	0.72	21.7
Euschistus quadrator (Rolston)	0.26	0.13
Euschistus ictericus (L.)	1.5	1.6
Euschistus obscurus (Palisot)	0.21	0.38
Euschistus servus (Say)	74.1	28.4
Euschistus tristigmus (Say)	22.5	45.9
Hymenarchys nervosa (Say)	0.07	0
Holocostethus limbolarius (Stål)	0.01	0.02
Menecles insertus (Stål)	0.02	0.02
Mormidea lugens (F.)	0.03	0
Nezara viridula (L.)	0.13	1.1
Oebalus pugnax (F.)	0.11	0.03
Piezodorus guildinii (Westwood)	0.005	0
Proxys punctulatus (Palisot)	0.03	0
Thyanta custator accerra (McAtee)	0.07	0.14

Supplemental Materials Table S2. Stink bug adult and nymph aggregation indices (I_a)[a] in Irwin County, GA, USA during 2016 based on SADIE spatial analysis.

	E. servus		*E. tristigmus*		*C. hilaris*		
Date	Adult	Nymph	Adult	Nymph	Adult	Nymph	Available host crop[b]
6/10/16	0.90	1.37	1.12	1.15	1.08	1.03	CO
6/17/16	0.80	0.88	1.12	**1.57**	0.75	0.89	CO
6/24/16	1.02	0.88	1.33	1.06	0.77	0.88	CO
7/1/16	1.02	1.02	0.92	0.81	-	0.80	CO
7/8/16	1.19	0.94	1.28	1.07	0.76	1.16	CO
7/15/16	1.22	1.03	**1.53**	1.38	1.03	1.13	CO, PE
7/22/16	1.07	0.92	1.27	1.33	1.08	0.85	CO, PE, CT
7/29/16	0.97	0.90	0.93	1.09	1.10	0.89	CO, PE, CT
8/5/16	0.99	1.22	1.37	0.90	1.67	0.94	PE, CT
8/12/16	1.16	0.96	1.33	1.07	1.45	0.78	PE, CT
8/19/16	1.28	1.30	1.22	0.94	1.41	1.17	PE, CT
8/26/16	1.06	0.98	1.28	1.37	0.97	**1.57**	PE, CT
9/2/16	1.07	1.03	1.33	1.16	-	**1.72**	PE, CT
9/9/16	1.01	1.01	0.91	1.38	1.39	1.04	PE
9/16/16	1.15	1.09	1.04	1.09	1.16	1.26	PE
9/23/16	1.52	0.95	1.26	1.40	1.23	1.07	
9/30/16	1.03	0.75	1.17	0.89	0.76	1.07	
10/7/16	1.19	1.29	1.32	0.78	1.00	0.84	
10/14/16	1.46	**1.45**	1.15	1.00	0.92	1.08	
10/21/16	**2.61**	1.36	1.02	0.86	1.27	1.26	
10/28/16	1.27	0.74	1.22	0.87	0.93	0.93	
11/4/16	1.05	0.95	1.14	0.75	0.91	1.09	
11/11/16	1.06	1.15	1.09	1.32	1.30	1.37	
11/18/16	0.96	1.27	**1.55**	1.15	0.79	**1.58**	
11/25/16	1.21	**1.94**	1.25	1.21	0.77	-	
12/2/16	0.97	1.06	1.11	1.01	-	-	
12/9/16	1.13	1.43	1.01	0.90	-	-	

[a] I_a values in bold indicate that $P_a < 0.05$.

[b] Peanut (PE), cotton (CT), and corn (CO).

Supplemental Materials Table S3. Stink bug adult and nymph aggregation indices (I_a)[a] in Irwin County, GA, USA during 2017 based on SADIE spatial analysis.

	E. servus		*E. tristigmus*		*C. hilaris*		
Date	Adult	Nymph	Adult	Nymph	Adult	Nymph	Available host crop[b]
3/20/17	**1.61**	1.02	1.26	-	1.39	0.73	
3/27/17	1.17	**1.53**	1.29	-	0.86	-	
4/3/17	**2.28**	-	1.37	-	-	-	
4/10/17	1.15	1.07	**1.47**	1.11	-	0.78	
4/17/17	0.99	-	1.26	0.92	0.78	-	
4/24/17	1.00	0.72	1.11	-	-	-	
5/1/17	1.20	1.03	0.87	1.15	-	0.76	
5/8/17	1.08	1.30	0.78	0.88	-	-	
5/15/17	1.13	**1.36**	1.05	1.28	1.08	1.08	
5/22/17	1.04	0.96	**1.49**	0.81	-	-	
5/29/17	1.00	0.89	1.08	1.41	-	0.93	
6/6/17	0.97	0.82	0.84	0.90	0.85	-	CO
6/12/17	1.01	0.80	0.93	1.18	0.75	0.78	CO
6/19/17	**1.52**	0.96	1.32	0.96	0.75	-	CO
6/26/17	**1.48**	0.89	1.17	1.27	-	1.15	CO
7/3/17	**1.45**	0.87	1.10	1.01	0.77	0.98	CO
7/10/17	1.07	0.84	1.31	0.96	-	-	CO
7/17/17	1.26	1.31	1.09	0.85	-	0.77	CO, PE
7/24/17	1.25	1.08	1.09	**1.42**	0.95	-	CO, PE, CT
7/31/17	**1.92**	1.06	1.23	0.86	0.83	0.75	CO, PE, CT
8/7/17	**2.36**	0.97	0.82	1.09	-	1.13	PE, CT
8/14/17	**1.43**	**1.57**	1.04	1.11	0.82	-	PE, CT
8/21/17	1.28	1.03	0.90	1.14	1.02	0.94	CT
8/28/17	1.27	0.95	0.95	1.07	0.77	0.77	CT

[a] I_a values in bold indicate that $P_a < 0.05$.

[b] Peanut (PE), cotton (CT), and corn (CO).

Supplemental Materials Table S4. Stink bug adult and nymph aggregation indices (I_a)[a] in Irwin County, GA, USA during 2018 based on SADIE spatial analysis.

	E. servus		*E. tristigmus*		*C. hilaris*		
Date	Adult	Nymph	Adult	Nymph	Adult	Nymph	Available host crop[b]
3/19/18	1.13	-	**1.48**	-	-	-	
3/26/18	1.26	-	1.36	-	-	-	
4/2/18	1.16	-	**1.43**	-	-	-	
4/9/18	1.27	-	1.02	-	-	-	
4/16/18	1.02	-	1.15	-	-	-	
4/23/18	0.84	-	1.15	-	-	-	
4/30/18	1.09	1.28	**1.57**	-	-	-	
5/7/18	0.84	0.82	**1.72**	1.05	-	-	
5/14/18	1.21	0.78	1.24	1.04	-	-	
5/21/18	1.11	0.82	1.32	0.96	-	-	
5/28/18	**1.74**	0.94	1.31	1.26	-	-	
6/4/18	1.16	0.75	**1.61**	1.29	-	-	CO
6/11/18	1.20	0.93	0.90	0.79	-	-	CO
6/18/18	1.26	1.2	1.12	**1.85**	-	**1.84**	CO
6/25/18	1.33	1.28	1.07	0.86	-	1.24	CO
7/2/18	**1.63**	0.96	1.09	1.05	0.72	1.34	CO
7/9/18	**2.23**	1.16	1.04	0.93	-	0.88	CO
7/16/18	**2.71**	**1.7**	1.27	**1.4**	0.75	0.94	CO
7/23/18	**2.03**	1.1	1.14	0.79	**1.62**	0.87	CO, PE
7/30/18	1.40	1.01	1.09	-	1.03	-	CO, PE, CT
8/6/18	0.85	1.22	1.04	0.86	0.76	**1.6**	PE, CT
8/13/18	0.86	0.91	1.26	1.04	0.94	1.2	PE, CT
8/20/18	1.06	1.26	**2.14**	1.2	0.77	-	PE, CT
8/27/18	1.26	0.93	1.04	0.94	1.12	1.23	PE, CT
9/3/18	1.16	1.05	1.26	1.05	**1.54**	1.28	PE, CT
9/10/18	1.20	1.41	**1.73**	0.94	0.82	1.17	PE, CT
9/17/18	1.11	**1.66**	**1.83**	0.97	0.76	1.14	PE
9/24/18	1.26	0.99	**2.13**	**1.78**	0.87	1.1	PE
10/1/18	1.16	0.99	1.32	**1.58**	0.87	0.95	
10/8/18	**1.76**	0.85	0.88	0.85	**1.93**	**1.64**	
10/15/18	**1.58**	0.82	1.01	0.77	**2.03**	**1.93**	
10/22/18	**1.51**	1.38	0.97	0.93	0.93	1.29	

[a] I_a values in bold indicate that $P_a < 0.05$.

[b] Peanut (PE), cotton (CT), and corn (CO).

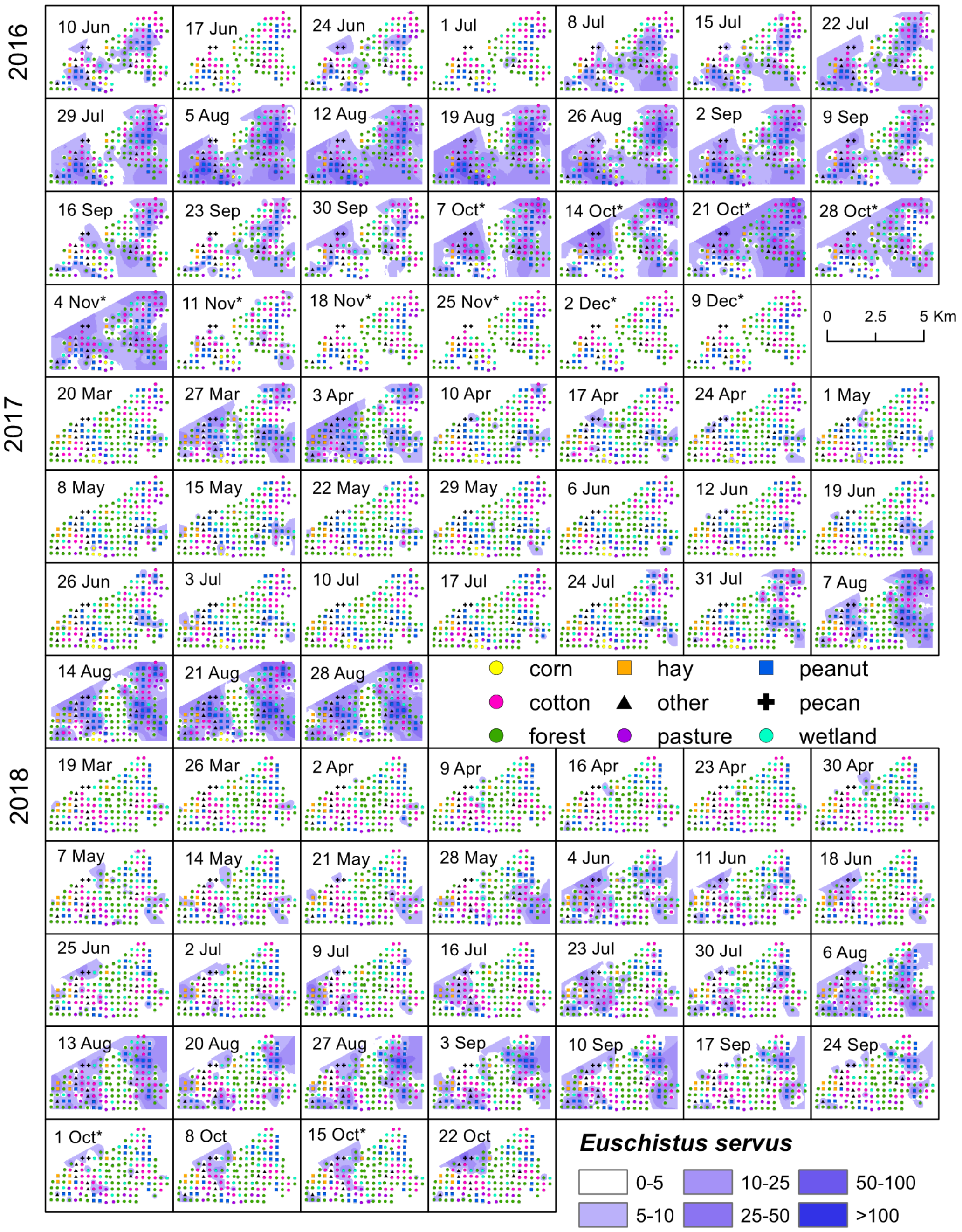

Supplemental Materials Figure S1. Interpolated maps based on annual trap capture that shows the distribution of the total number of *Euschistus servus* (nymphs and adults) captured from 2016-2018. An asterisk following sample dates indicates that adults in overwintering condition were captured.

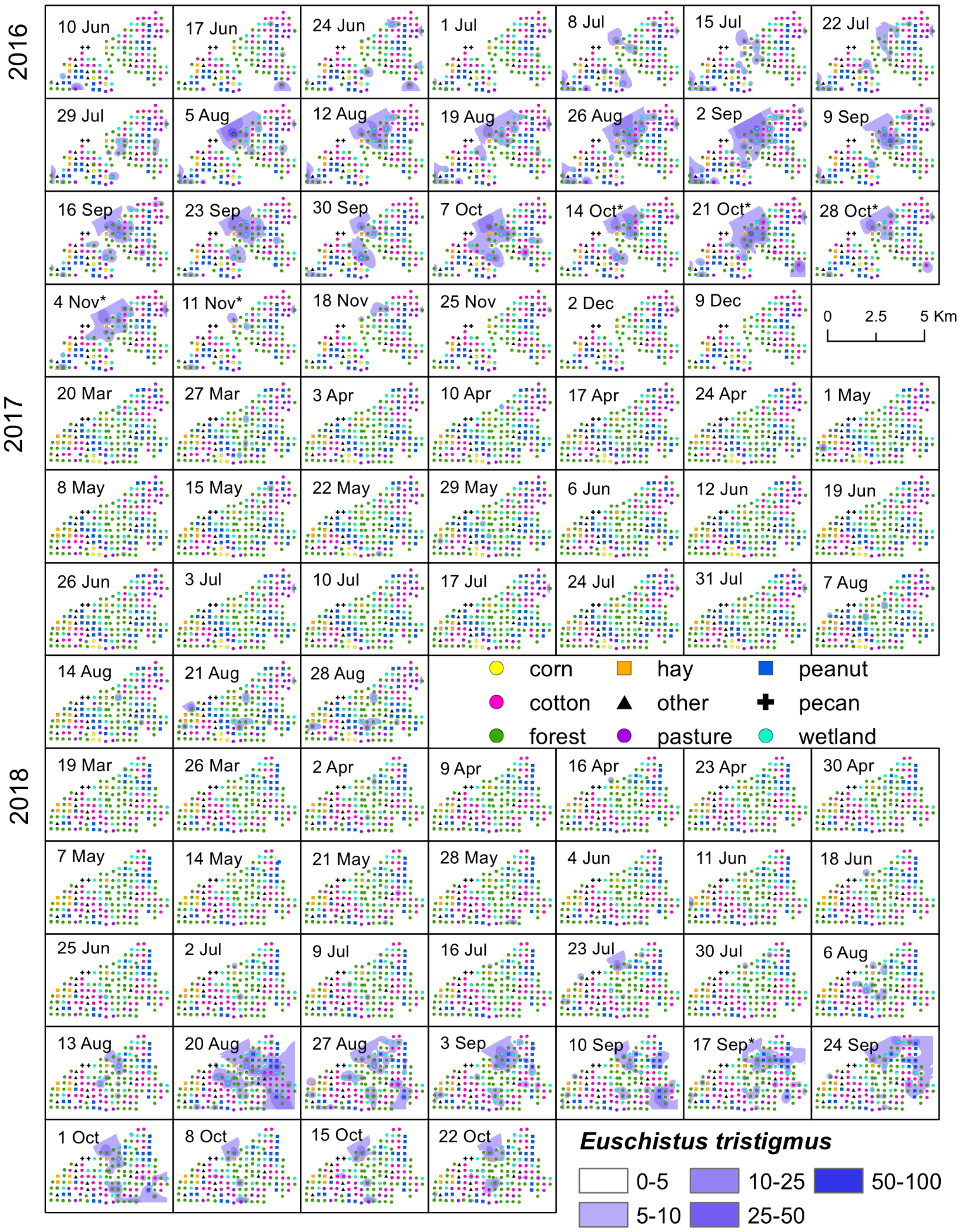

Supplemental Materials Figure S2. Interpolated maps based on annual trap capture that shows the distribution of the total number of *Euschistus tristigmus* (nymphs and adults) captured from 2016-2018. An asterisk following sample dates indicates that adults in overwintering condition were captured.

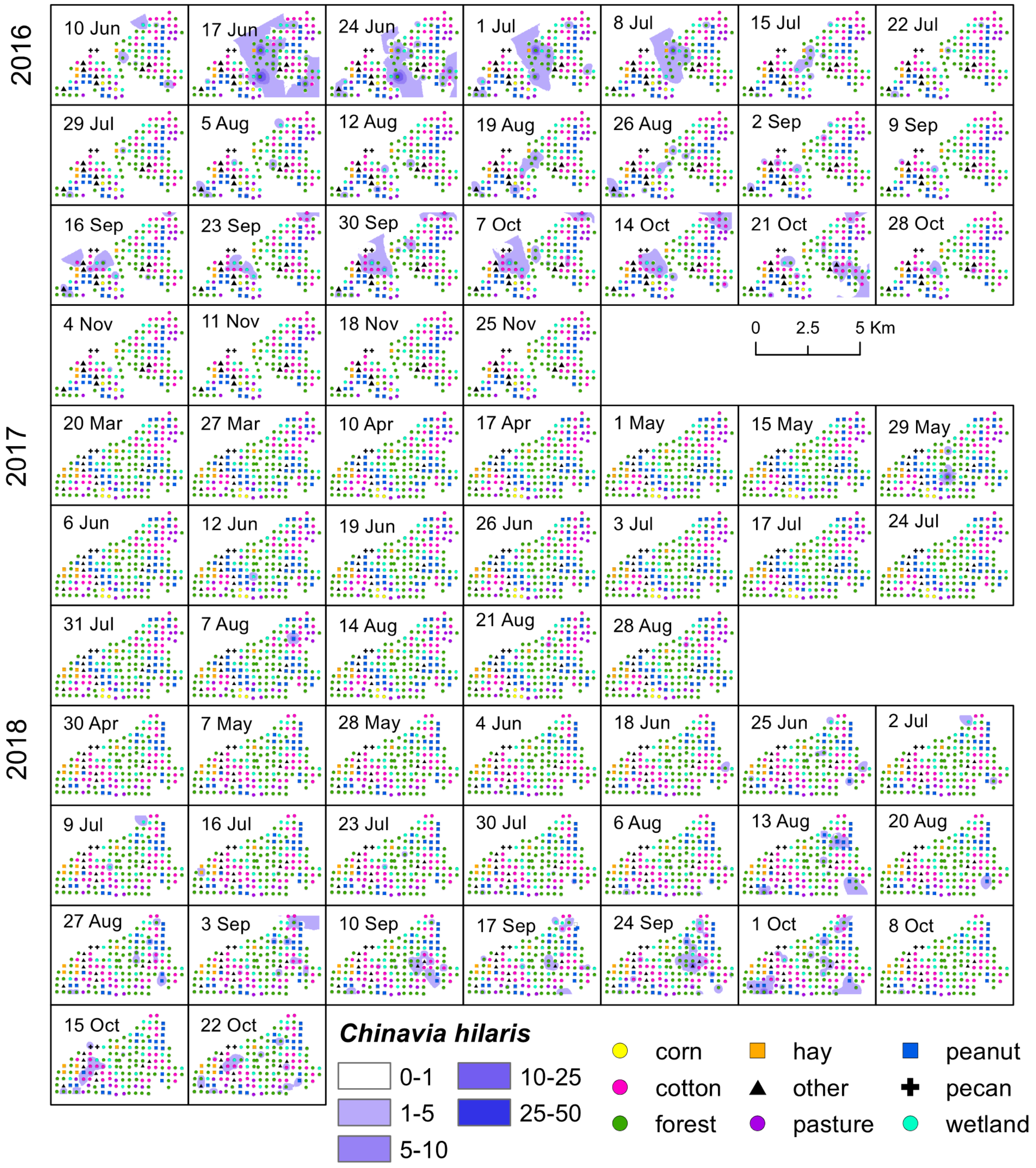

Supplemental Materials Figure S3. Interpolated maps of *C. hilaris* distributions from 2016-2018. Sample weeks were omitted from the map if no *C. hilaris* adults or nymphs were captured.
